# The Antihyperglycemic Effects of Rhizoma Coptidis and Mechanism of Actions: A Review of Systematic Reviews and Pharmacological Research

**DOI:** 10.1155/2014/798093

**Published:** 2014-04-10

**Authors:** Hui Wang, Wei Mu, Hongcai Shang, Jia Lin, Xiang Lei

**Affiliations:** Tianjin Institute for Clinical Evaluation of Traditional Chinese Medicine, Tianjin University of Traditional Chinese Medicine, No. 88 Yuquan Road, Nankai District, Tianjin 300193, China

## Abstract

Rhizoma Coptidis (Huang Lian in Chinese pinyin) is among the most widely used traditional Chinese herbal medicines and has a profound history of more than 2000 years of being used as a therapeutic herb. The antidiabetic effects of Rhizoma Coptidis have been extensively investigated in animal experiments and clinical trials and its efficacy as a promising antihyperglycemic agent has been widely discussed. In the meantime, findings from modern pharmacological studies have contributed the majority of its bioactivities to berberine, the isoquinoline alkaloids component of the herb, and a number of experiments testing the antidiabetic effects of berberine have been initiated. Therefore, we conducted a review of the current evidence profile of the antihyperglycemic effects of Rhizoma Coptidis as well as its main component berberine and the possible mechanism of actions, in order to summarize research evidence in this area and identify future research directions.

## 1. Introduction

Diabetes mellitus refers to a metabolic disorder of multiple etiology characterized by chronic hyperglycaemia with disturbances of carbohydrate, fat, and protein metabolism resulting from disturbed insulin secretion, insulin action, or both [1]. There are two possible types of diabetes mellitus. Type 1 diabetes, also known as insulin-dependent diabetes, results from an absolute lack of insulin due to autoimmune destruction of the insulin-producing beta cells in the pancreas [2]. Type 2 or non-insulin-dependent diabetes is a metabolic disorder characterized by insulin resistance, relative insulin deficiency, and hyperglycemia [3]. Diabetes mellitus is most closely related to the wasting (Xiao Ke in Chinese pinyin) syndrome as defined by the traditional Chinese medicine diagnostic pattern. Patients with this syndrome typically experience clinical manifestations of emaciation (Xiao in Chinese) and thirst (Ke in Chinese).

According to the WHO diabetes fact sheets, 347 million people in the world have diabetes [4]. In 2004, an estimated 3.4 million people worldwide died from consequences of high blood sugar, and ca. above 80% of diabetes deaths happen in underdeveloped countries [4]. Facing this stark reality, traditional Chinese herbal remedy such as Rhizoma Coptidis (Huang Lian in Chinese pinyin), with its long proven effects for a number of chronic diseases in clinical application and relatively low cost, has been broadly investigated in Asian countries for potential antihyperglycemic effects.

Rhizoma Coptidis (Huang Lian) is the dried rhizome of* Coptis chinensis* Franch,* Coptis deltoidea* C. Y. Cheng et Hsiao or* Coptis teeta* Wall. As first recorded in* Shennong's Materia Medica *in the eastern Han dynasty (25–220 AD), the herbal medicine has been prescribed by Chinese herbalists for a variety of illnesses and conditions for more than 2000 years. According to traditional beliefs, Rhizoma Coptidis is cold in nature and bitter in taste and enters the heart, spleen, stomach, liver, gallbladder, and large intestine meridians. It has the function of clearing heat, drying dampness, and purging fire toxins [5]. Main indications include the wasting (Xiao Ke) syndrome, distention, and fullness due to dampness and heat, sickness, acid regurgitation, jaundice, palpitation, diarrhea caused by bacterial infection, high fever, heart-fever hyperactivity, restlessness and insomnia, blood spitting or nose bleeding due to extra heat in the blood, red eyes, and toothache [5].

As early as the Wei and Jin dynasties in the Chinese history (220–589 AD), Rhizoma Coptidis was described as a therapeutic agent for patients suffering from the wasting syndrome. In dynasties that followed, numerous records had been kept in a series of herbal classics of the medicinal use of Rhizoma Coptidis for the wasting syndrome, either alone or combined with other herbs in a formula. This proved the prevalence of the use of Rhizoma Coptidis since ancient times and formed the empirical evidence base for its antidiabetic effects. For example, in the* Miscellaneous Records of Famous Physicians*, compiled around 510 AD, Rhizoma Coptidis was first described as an agent for the wasting syndrome [6]. In the* Newly Revised Materia Medica* compiled during the Tang dynasty (618–907 AD), it was noted that “Huang lian grown in west China is bulky, bitter and good for treating the wasting syndrome” [7]. An analytical review of the Song dynasty (960–1279 AD) medical formulary* Formulas from Benevolent Sages* found that Rhizoma Coptidis was among the top ten most frequently used medicinal herbs in formulas designated for the wasting syndrome [8]. Furthermore, thirteen among the total of 64 herbal formulas for treating the wasting syndrome collected in the* Puji Fang Prescriptions for Universal Relief*, completed around 1406 AD in the Ming dynasty, contained Rhizoma Coptidis [9]. In the most comprehensive medical book of traditional Chinese medicine, the* Compendium of Materia Medica*, published in the same dynasty, it recorded that “Huang lian, steamed with wine, is used for treating emaciation, thirst and excessive excretion of urine” [9].

Modern pharmacological research identified the major chemical constituents of Rhizoma Coptidis to be alkaloids including berberine, coptisine, worenine, palmatine, jatrorrhizine, and epiberberine [10, 11]. Among the many constituents, the berberine component is generally considered the primary contributor to its main bioactivities such as the antibiotic, antioxidant, and anti-inflammatory properties [12]. In recent years, berberine and Rhizoma Coptidis extracts have also been reported to have multiple antidiabetic activities such as regulating lipid, balancing glucose metabolism, and improving insulin resistance, and the underlying mechanisms of action have been extensively investigated [12].

In view of this, a comprehensive review of the relevant literatures on the antihyperglycemic effects of Rhizoma Coptidis (or berberine) and the mechanism of actions was conducted. The aim of this study is to give a summary of the existing evidence.

## 2. Method

In January 2013 two reviewers (Hui Wang and Wei Mu) searched the following Chinese-language electronic databases: Chinese Biomedical Literature Database (CBM, 1980–2013), Chinese Journal Full-Text Database (CNKI, 1980–2013), Weipu Journal Database (VIP, 1989–2013), and Wanfang Data (1990–2013), and three English-language databases: PubMed, EMBASE (1989–2013), and the Cochrane Library. The search terms included “huang lian,” “Coptis,” “berberine,” “hypoglycemic,” “diabetes,” and “xiaoke” in English or Chinese. These terms were searched as free text in the title or the abstract.

Two reviewers Hui Wang and Wei Mu screened citations identified from electronic searches and retrieved the full texts of relevant studies. Then they summarized records in ancient medical books and the findings of systematic reviews and pharmacological studies on the antihyperglycemic effects of berberine, Rhizoma Coptidis extracts, and other Rhizoma Coptidis-containing agents.

## 3. Results

### 3.1. Overview of Systematic Reviews

Two systematic reviews on the antihyperglycemic effects of berberine were identified. In the first study authored by Dong et al. [13], a total of fourteen randomized controlled trials (RCTs) were included and the results of these studies were subjected to meta-analysis and subgroup analysis. In the later systematic review by Narenqimuge et al. [14], the results of the included ten RCTs were reported descriptively due to significant statistical heterogeneity across studies. The characteristics and results of these two systematic reviews were presented in Table 1.

Drawn upon the above research findings, the isoquinoline-type alkaloid berberine has beneficial effects for blood glucose control in the treatment of type 2 diabetic patients and exhibits efficacy comparable with that of conventional oral hypoglycaemics. No significant statistical difference in the incidence of adverse events was observed between groups. However, the evidence is inconclusive because clinical trials included in the two systematic reviews were of low methodological quality. Therefore, the antihyperglycemic effects of berberine warrant further examination and more rigorously controlled, methodologically sound, and scientifically designed RCTs need to be conducted.

### 3.2. Review of Pharmacological Research

A number of animal experiments investigating the antihyperglycemic effects of Rhizoma Coptidis, berberine, or herbal prescriptions in which Rhizoma Coptidis plays a dominant role were identified through electronic searches and careful screening. A summary of the experimental models used and the antidiabetic mechanism of berberine observed in these studies were presented in Table 2. From this table, we found the antidiabetic efficacy of berberine associated most closely with its ability to improve insulin sensitivity, influence insulin secretion, and regulate carbohydrate metabolism, and a majority of the pharmacological experiments focused on these aspects [15–61]. Moreover, some additional bioactivities of berberine which may facilitate its antidiabetic effects were identified, such as its antioxidant, lipid regulatory, and anti-inflammatory functions as well as its renoprotective properties to prevent diabetes complications. These effects of berberine and the relevant mechanisms were demonstrated in Table 3.

Findings from previous researches showed that the insulin-stimulated glucose uptake by target tissues such as adipocytes and skeletal muscles involved a series of signaling transduction cascades starting from insulin receptor (InsR) via insulin receptor substrate-1 (IRS-1) and phosphatidylinositol 3-kinase (PI-3K) and leading to the translocation of glucose transporter (GLUT4) [77]. From this comprehensive review of the existing pharmacological research on the antihyperglycemic effects of Rhizoma Coptis and its major chemical ingredient berberine, the reviewers summarized a variety of possible mechanisms of action behind its antidiabetic properties, which include the promotion of insulin secretion and release, reparation of pancreatic islets *β*-cells, enhancement of insulin sensitivity, suppression of gluconeogenesis in the liver, promotion of glucose disposal in the periphery, and inhibition of aldose reductase [15–61]. The mechanisms for Rhizoma Coptis and its component berberine's other bioactivities that may facilitate its antidiabetic functions include ameliorating oxidative stress accompanying diabetes, regulating plasma levels of adiponectin and other relevant inflammatory factors, increasing adipocytes glucose transportation and consumption, and modulating metabolism-related protein expression [62–76].

In addition to the above findings, results from a few studies [78–89] investigating the effects of Rhizoma Coptidis extracts, Rhizoma Coptidis-dominant couplet medicines, and Rhizoma Coptidis-containing Chinese patent drugs have showed them all to possess certain antihyperglycemic effects. Rhizoma Coptidis-dominant couplet medicines included Rhizoma Coptidis coupled with Panax Ginseng, Rhizoma Coptidis coupled with dried Rehmannia root, and a combination of Rhizoma Coptidis, Astragalus Mongholicus, and Solomonseal Rhizome. Rhizoma Coptidis-containing Chinese patent drug involved Jinlian Jiangtang capsules and Jinqi Jiangtang tablets. Their therapeutic properties, possible mechanisms, and other information were listed in Table 4.

## 4. Discussion

According to traditional beliefs, Chinese herbal remedy helps recover inner peace and tranquility in the human body with its multiple active constituents taking effects through various mechanisms and pathways. Therefore, the use of Chinese herbal medicines for diabetes treatment or for the prevention of diabetes complications might be generally considered good for the patients' general well-being, apart from their effectiveness and safety.

The existing evidence profile of the antihyperglycemic effects of Rhizoma Coptidis includes both textual records in ancient herbal classics and findings from animal experiments and systematic reviews of RCTs. Modern research uniformly focuses on berberine, whereas the pharmacological actions of other active ingredients of Rhizoma Coptidis, of single herb remedy, and of Rhizoma Coptidis-dominant couplet medicines and Rhizoma Coptidis-containing patent drug still remain to be investigated.

As was summarized in this review, the antihyperglycemic effects of Rhizoma Coptidis may rely upon drug actions on a variety of targets via multiple pathways. Many animal experiments [15–76, 78–89] have proposed the scientific rationale for Rhizoma Coptidis, Rhizoma Coptidis-containing agents, or its major component berberine's antihyperglycemic effects by identifying possible mechanisms of actions. The widespread use of Rhizoma Coptidis as a routine clinical treatment for diabetes is promising because there is abundant supply, the herb is relatively inexpensive, and it has a good safety profile [13, 14]. However, the results of both systematic reviews included in this study need to be interpreted with caution. As large-scale, rigorously controlled, and multicenter randomized controlled clinical studies are still lacking, the clinical efficacy and safety of Rhizoma Coptidis and berberine for antidiabetic use needs further investigation.

Furthermore, there were other issues to consider before Rhizoma Coptidis can be put into extensive clinical use. For instance, the most appropriate drug form and dosage, dose-effect relationship, and drug-drug interactions should be made clear through a series of pharmacological experiments and long-term clinical observations. Also, whether the antihyperglycemic effect is best exerted synergically in a prescription or independently as an active component remains to be investigated. Besides, the possible antidiabetic effects of the other chemical ingredients of Rhizoma Coptidis and the interactions among its various components, as well as the long-term health benefits of its use in diabetic patients, are all problems that need to be addressed in future research.

## Figures and Tables

**Table 1:** A summary of findings from systematic reviews on the antihyperglycemic effects of berberine.

Outcomes	Study ID	Study Year	Number of RCTs	Number of cases	Results	Conclusion
FPG/PPG/HbA1c/FINS/TG/TC/LDL-C/HDL-C	Dong et al. [13]	2012	4	271	Compared with lifestyle changes with or without placebo, berberine plus lifestyle changes showed significantly better hypoglycaemic and antidyslipidemic effects.	Trials had poor methodological and reporting quality. The conclusions made were inconclusive and further research is needed.
FPG/HbA1c/TC/LDL-C	Dong et al. [13]	2012	7	448	Compared with oral hypoglycaemics (metformin, glipizide, or rosiglitazone) berberine did not demonstrate significantly better hypoglycaemic effects but showed mild antidyslipidemic effects.	
FPG/PPG/HbA1c/FINS/TG/TC/LDL-C/HDL-C	Dong et al. [13]	2012	6	396	Compared with oral hypoglycaemic drugs, berberine combined with the same oral hypoglycaemics can better control blood sugar in the patients.	

FBG/2hPBG/HbA1c/LDL-C/HDL-C/TG/TC/BMI	Narenqimuge et al. [14]	2012	10	647	Berberine was effective in lowering FBG but not better than metformin, glipizide, or rosiglitazone. Berberine had no proven effects in decreasing PBG, HbAlc, or BMI and in regulating lipid metabolism.	Trials were at high risk of bias. High-quality trials are needed.

Adverse events	Dong et al. [13]	2012	14	1068	Three studies did not report AEs. Three reported no AEs. Three reported adverse events but did not indicate the group in which they occurred. Five reported AEs in the berberine group.	No significant difference between the treatment and the control group.

Adverse events	Narenqimuge et al. [14]	2012	7	435	Seven studies reported that AEs happened in the course of the treatment, mostly gastrointestinal reactions such as constipation and diarrhea. Of them 3 studies reported the number of cases of AEs.	No significant difference between the treatment and the control group.

FPG: fasting blood glucose; PPG: postprandial plasma glucose; HbA1c: hemoglobin A1c; TG: triglyceride; TC: total cholesterol; LDL-C: low-density lipoprotein; HDL-C: high-density lipoprotein; FINS: fasting insulin; 2hPBG: 2-hour postprandial blood glucose; BMI: body mass index; AEs: adverse events.

**Table 2:** Summary of berberine's effects on insulin and glucose metabolism and the mechanism of action [15–61].

Experimental model	Mechanism of action
T2DM Chinese hamsters	Changed the expression of hepatic peroxisome proliferator-activated receptors and its target genes [15]
3T3-L1 cells	Increased glucose uptake in fat cells and inhibited the differentiation of preadipocytes [16]
Sprague Dawley rats	Reduced plasma FFA and triglyceride levels and inhibited the expression of liver TNF-*α* [17]
Sprague Dawley rats	Increased HNF-4*α* expression [18]
Wistar rats	Regulated the expression of endoplasmic reticulum chaperone ORP150 and reduced ER stress [19]
HepG2 cells	Regulated AMPK activity to decrease its downstream gluconeogenesis protein expression [20]
Wistar rats	Inhibited pancreatic *β*-cell apoptosis by inhibiting ASK1 protein expression [21]
Wistar rats	Increased PI-3K p85 and GLUT4 protein expression in skeletal muscles of T2DM rats [22]
Insulin resistant rat models	Inhibited TNF-*α* secretion and reduced serum free fatty acid level [23]
Wistar rats	Increased mRNA expression of adiponectin gene and decreased IRI in T2DM rats [24]
T2DM Chinese hamsters	Inhibited the expression of PEPCK, 6Pase, and PGC-1*α* by enhancing CYP7A1 and Gck expression induced by the upregulation of LXR*α* expression [25]
L6 myotubes	Inhibited fatty acid uptake at least in part by reducing PPAR gamma and FAT/CD36 expression [26]
L6 rat skeletal muscle cells	Induced InsR gene expression through a protein kinase C (PKC) dependent activation of its promoter. Inhibited PKC abolished BBR-caused InsR promoter activation and InsR mRNA transcription [27]
Nonalcoholic fatty liver disease rat liver	Upregulated the mRNA and protein levels of IRS-2 [28]
3T3-L1 adipocytes	Reversed free fatty acid-induced insulin resistance in 3T3-L1 adipocytes through targeting IKK*β* [29]
Cultured HepG2 cells	Attenuated ER stress and improved insulin signal transduction [30]
Rat skeletal muscle cells	Modulated key molecules in the insulin signaling pathway, leading to increased glucose uptake in insulin-resistant cells [31]
Insulin resistant rat models	Stimulated AMPK activity [32]
T2DM hamsters	Altered the transcriptional programs of the visceral white adipose tissue LXRs, PPARs, and SREBPs [33]
Diabetic hamsters	Altered the transcriptional programs of hepatic SREBPs, LXR*α*, and PPAR*α* [34]
Mouse primary hepatocyte	Upregulated HNF4*α* expression to induce hepatic glucokinase activity [35]
Mouse primary hepatocyte	Upregulated HNF6 mRNA expression and induced hepatic glucokinase activity [36]
HepG2 cells	Increased hepatic glucose consumption [37]
Wistar rats	Elevated IRS-1, IRS-2, and p85 mRNA expression in the peripheral tissues [38]
Sprague Dawley rats	Increased the content of GLU4 mRNA in skeletal muscles, increased the content of GLUT4 protein in cells, and enhanced insulin activity in the peripheral tissues [39]
Kunming mice	Inhibited gluconeogenesis and/or stimulated glycolysis [40]
Diabetic rat model	Stimulated GLP-1 release [41]
Wistar rats	Regulated INS and GH levels by enhancing SS levels through the hypothalamus-pituitary-pancreatic axis system [42]
HepG2 cells	Increased InsR mRNA transcription and protein expression [43]
Alloxan-induced diabetic mice	Upregulated the activity of Akt [44]
Wistar rats	Stimulated GK activity and expression [45]
3T3-L1 adipocytes and L6 myocytes	Inhibited PTP 1B activity and increased phosphorylation of IR, IRS1, and Akt in 3T3-L1 adipocytes [46]
Streptozotocin-induced diabetic rats	Enhanced GLP-1-(7-36) amide secretion [47]
Mammalian cells	Functioned as an agonist of the fatty acid receptor GPR40 [48]
T2DM rat models	Lowered serum RBP4 levels and upregulated the expression of tissue GLUT4 protein [49]
Streptozotocin-induced diabetic rats	Exhibited inhibitory effects on intestinal disaccharidases and *β*-glucuronidase [50]
L6 rat skeletal muscles	Stimulated glucose uptake through the AMP-AMPK-p38 MAPK pathway [51]
3T3-L1 adipocytes	Enhanced GLUT1 expression and stimulated the GLUT1-mediated glucose uptake by activating GLUT1 [52]
Alloxan-induced diabetic C57BL/6 mice	Upregulated Akt activity via insulin signaling pathways [53]
Molecular model	Inhibited H-PTP 1B [54]
Streptozotocin-induced diabetic rats	Involved PKA-dependent pathways [55]
Normal animals (dogs and rats)	Acutely inhibited *α*-glucosidase [56]
Molecular model	Inhibited DPP IV [57]
L929 fibroblast cells	Significantly activated GLUT1 transport [58]
Diabetic rats	Directly inhibited gluconeogenesis in the liver [59]
Rat model	Inhibition of glucose oxidation in mitochondria may contribute to increased AMP/ATP ratio and AMPK activation [60]
Caco-2 cell line	Inhibited *α*-glucosidase activity and decreased glucose transport across the intestinal epithelium [61]

T2DM: type 2 diabetes mellitus; TNF: tumor necrosis factor; FFA: free fatty acid; HNF: hepatocyte nuclear factor; ER: endoplasmic reticulum; ORP: oxygen-regulated protein; AMPK: AMP-activated protein kinase; ASK: apoptosis signal-regulating kinase; mRNA: messenger RNA; IRI: insulin resistant index; PEPCK: phosphoenolpyruvate carboxylase kinase; PGC-1*α*: peroxisome proliferator-activated receptor-*γ* coactivator 1*α*; CYP7A1: cholesterol 7 *α*-hydroxylase; GCK: glucokinase; PI-3K: phosphatidylinositol 3-kinase; GLUT4: glucose transporter type 4; PPAR: peroxisome proliferator-activated receptor; FAT/CD36: fatty acid translocase; PKC: protein kinase C; BBR: berberine; IKK*β*: inhibitor kappa B kinase *β*; LXRs: liver X receptors; SREBPs: sterol regulatory element binding proteins; IRS: insulin receptor substrates; GLP: glucagon-like peptide; INS: insulin; GH: growth hormone; SS: somatostatin; Akt: protein kinase B; PTP1B: protein tyrosine phosphatase 1B; IR: insulin resistance; GPR40: G protein-coupled receptor 40; RBP4: retinol-binding protein 4; H-PTP 1B: human protein tyrosine phosphatase 1B; DPP IV: dipeptidyl peptidase IV; AMP: adenosine monophosphate; ATP: adenosine triphosphate.

**Table 3:** Summary of other effects of berberine and the mechanism of action [62–76].

Effects	Experimental model	Mechanism of action
Antioxidative effects	Type 2 diabetic rats	Reduced oxidative stress [62]

Hypolipidemic effects	Wistar rats	Decreased blood FFA level and enhanced the activity of lipoprotein lipase [63]
	Type 2 diabetic rats	Increased PPARs and P-TEFb mRNA and protein expression in the adipose tissue. Restored SOD and LPL activity and normalized malondialdehyde, FFA, TNF-*α*, and adiponectin levels [64]
	3T3-L1 adipocytes	Increased glucose transport and consumption in 3T3-L1 adipocytes [65]
	3T3-L1 adipocytes	Modulated metabolism-related PPARs expression and differentiation-related P-TEFb expression in adipocytes [66]
	3T3-L2 adipocytes	Activated adenosine monophosphate, activated protein kinase [67]
	Diabetic hyperlipidemic and normal rats	Modulated metabolism-related PPAR alpha/delta/gamma protein expression in the liver [68]

Anti-inflammatory actions	Type 2 diabetic rats	Regulated serum levels of inflammatory factors such as CRP, IL-6, TNF-*α*, and adiponectin [69]

Effects on renal injury	Glomerular mesangial cells	Inhibited NF-*κ*B activation and the expression of its downstream inflammatory factors to improve ECM accumulation and alleviate inflammatory injury in diabetic kidney [70]

Prevention of diabetes complications	Type 2 diabetic rats	Enhanced vascular smooth muscle activity [71]

Renal protective effects	Rat glomerular mesangial cells	Reduced the accumulation of extracellular matrix components including fibronectin and prevented the activation of the p38 MAPK signaling pathway [72]
	Diabetic rats	Inhibited glycosylation and exhibited antioxidative effects [73]
	Diabetic C57BL/6 mice	Deactivated the SphK-S1P signaling pathway [74]
	Streptozotocin-induced diabetic rats	Reduced oxidative stress and deactivated aldose reductase [75, 76]

SOD: superoxide dismutase; LPL: lipoprotein lipase; P-TEFb: positive transcription elongation factor b; CRP: C-reactive protein; IL-6: interleukin-6; NF-*κ*b: nuclear transcription factor-*κ*b; SphK: sphingosine kinase.

**Table 4:** Summary of the antihyperglycemic effects of Rhizoma Coptidis extracts, Rhizoma Coptidis-dominant couplet medicines, or Chinese patent drug and the relevant mechanisms [78–89].

Drug	Effects	Experimental model	Mechanism of actions
Rhizoma Coptidis decoction	Antihyperglycemic effects	Rat brain homogenate	Inhibited pancreatic lipid peroxidation [78]
	Reverse insulin resistance	Rat model with metabolic syndrome	Reversed insulin resistance, reduced visceral fat, and upregulated the expression of p-AMPK-A protein [79]

Water extracts of Rhizoma Coptidis	Reverse insulin resistance	3T3-L1 preadipocytes	Increased glucose uptake in preadipocytes [80]

Rhizoma Coptidis coupled with Panax Ginseng	Enhance insulin sensitivity	Type 2 diabetic rats	Lowered TNF-*α* levels, prevented TNF-*α* from inhibiting the expression of GLUT4 in fat and muscle cells, improved autophosphorylation of insulin receptor, inhibited second messenger activation, and promoted lipolysis [81]
	Reverse insulin resistance and regulate glucose and lipid metabolism	Type 2 diabetic rats	Reduced lipotoxicity [81]
	Antihyperglycemic effects	Type 2 diabetic rats	Activated PPAR-*γ*, slowed the release of FFA, enhanced the sensitivity of skeletal muscles and the liver to insulin, reduced the exportation of glycogen, and promoted the uptake of glucose in skeletal muscles [81]
	Reverse insulin resistance	Type 2 diabetic rats	Regulated FBG, INS, FFA, and TNF-*α* levels and improved insulin resistance [82]
	Enhance insulin sensitivity	Type 2 diabetic rats	Regulated lipid metabolism and reversed lipotoxicity [83]

A combination of Rhizoma Coptidis, Astragalus Mongholicus, and Solomonseal Rhizome	Vascular-protective	Wistar rats	Increased erythrocyte SOD activity and decreased serum MDA level, thereby reducing free radical damages in the hyperglycemic state [84]

Sanhuang compound (a mixture of Rhizoma Coptidis, Radix Astragali, and Radix Rehmanniae)	Antihyperglycemic and hypolipidemic effects	Type 2 diabetic rats	Protected and repaired pancreas, enhanced insulin secretion and glycogenesis, and inhibited gluconeogenesis [85]

Rhizoma Coptidis coupled with dried Rehmannia root	Reverse insulin resistance	Type 2 diabetic rats	Decreased the levels of inflammatory cytokine to improve insulin resistance and inhibited apoptosis of islet cells to protect islet *β*-cells [86]

Jinlian Jiangtang capsules	Antihyperglycemic effects	Kunming mice	Promoted insulin release and glucose uptake and improved *β*-cell functioning [87]

Jinqi Jiangtang tablets	Reverse insulin resistance and antihyperglycemic effects	Wistar rats	Inhibited resistin gene expression [88]
	Antihyperglycemic effects	3T3-L1 cells and KK-Ay mice	Activated the AMPK signaling pathway [89]

MDA: malondialdehyde.
